# Supplementary material for: The Important Role of m6A-Modified circRNAs in the Differentiation of Intramuscular Adipocytes in Goats Based on MeRIP Sequencing Analysis
Source: Int J Mol Sci. 2023 Mar 2;24(5):4817. doi: 10.3390/ijms24054817 (PMC10003525; doi:10.3390/ijms24054817)
Supplement: Supplementary file 1 [file ijms-24-04817-s001.zip › ijms-2146702-supplementary.pdf]

Supplementary Materials

The important role of m6A modified circRNAs in the differentiation of Intramuscular adipocyte in goats based on MeRIP sequencing analysis

Jianmei Wang [1,2,3], Xin Li [1,2,3], Wuqie Qubi [1,2,3], Yanyan Li [1,2,3], Yong Wang [1,2,3], Youli Wang [1,2,3] and Yaqiu Lin [1,2,3]

1 Key Laboratory of Qinghai-Tibetan Plateau Animal Genetic Resource Reservation and Utilization of Education Ministry, Southwest Minzu University, Chengdu 610041, China
2 Key Laboratory of Qinghai-Tibetan Plateau Animal Genetic Resource Reservation and Exploitation of Sichuan Province, Southwest Minzu University, Chengdu 610041, China
3 College of Animal & Veterinary Science, Southwest Minzu University, Chengdu 610041, China
* Correspondence: linyq1999@163.com

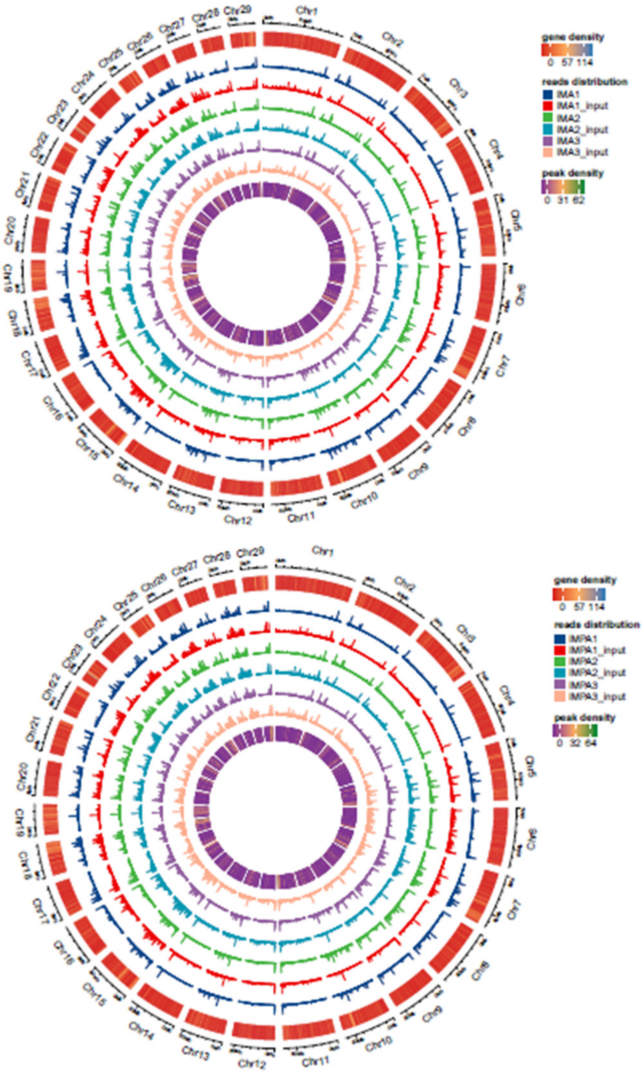

Figure S1. The distribution region of clean reads mapped to the reference genome. The circle map is in order from the outside to the inside. The outer circle is the genome length scale, the second outer circle is the gene density heat map of the species genome, the inner circle is the peak density heat map of the group of samples, and the rest of the circles are histograms of the distribution of reads of each sample on the genome.

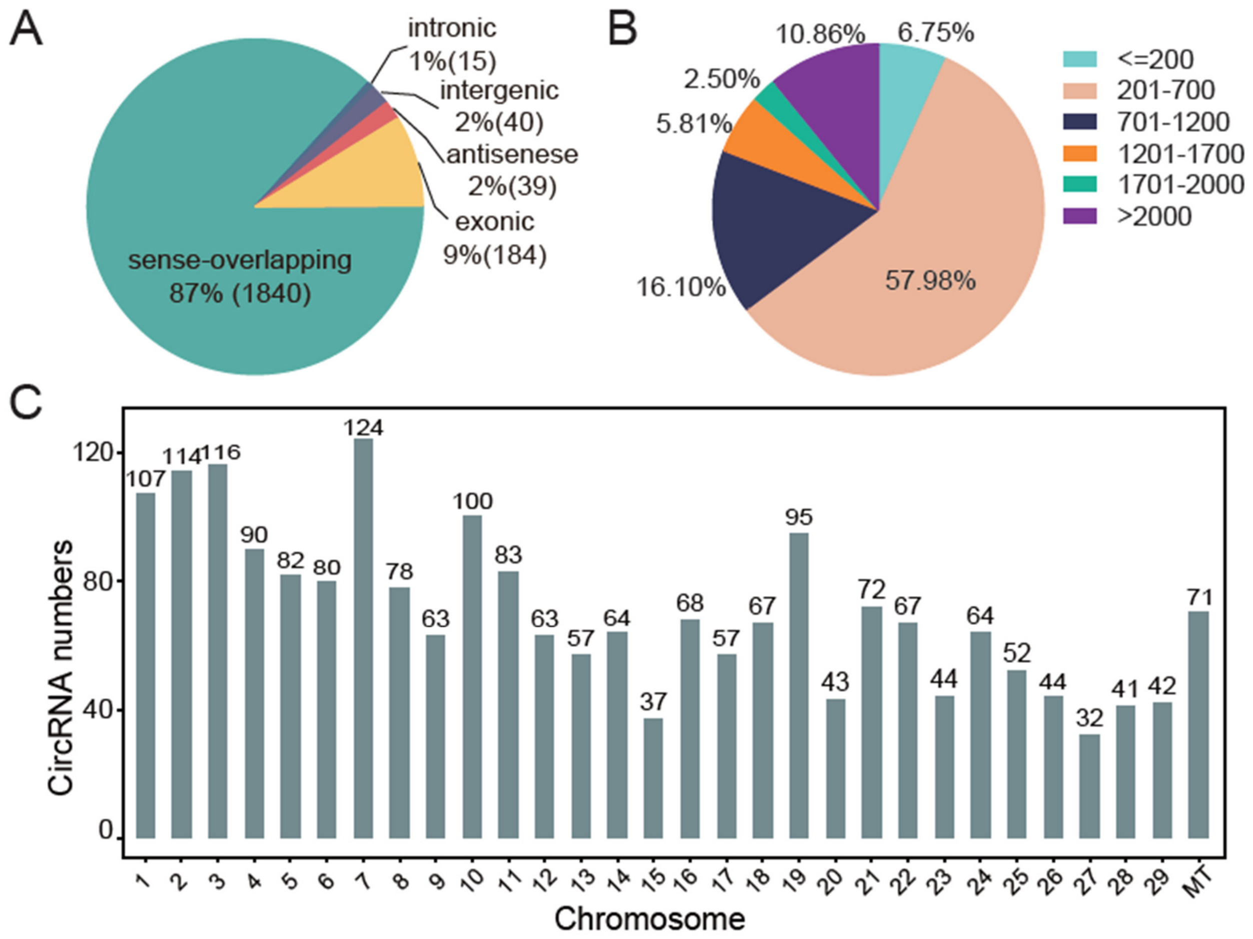

Figure S2. Overview of circRNAs in IMPA and IMA group. (A) The source of circRNAs in the two groups. (B) The length of circRNAs in the two groups. (C) Chromosome distribution.

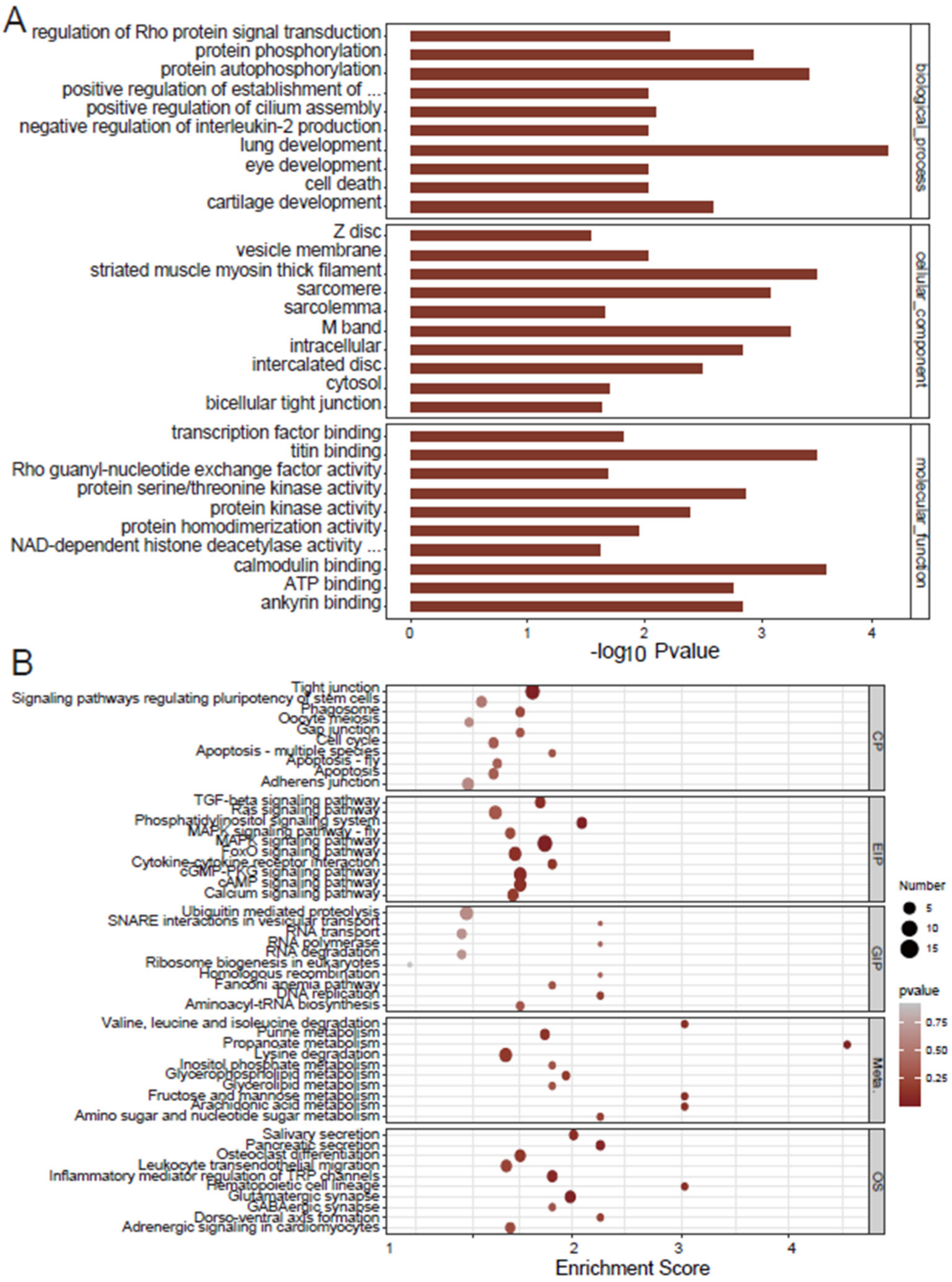

Figure S3. GO and KEGG enrichment analyses of differentially expressed circRNA in goat different adipocytes. (A) GO annotation of differential circRNA in two group. (B) KEGG enrichment scatter plot of differential circRNA in two group.

Table S1. The differential m6A methylation peaks between the IMA and IMPA groups.

Table S2. The m6A-circRNAs/miRNA interactions.
