# Supplementary figures and images for: The Important Role of m6A-Modified circRNAs in the Differentiation of Intramuscular Adipocytes in Goats Based on MeRIP Sequencing Analysis
Source: Int J Mol Sci. 2023 Mar 2;24(5):4817. doi: 10.3390/ijms24054817 (PMC10003525; doi:10.3390/ijms24054817)

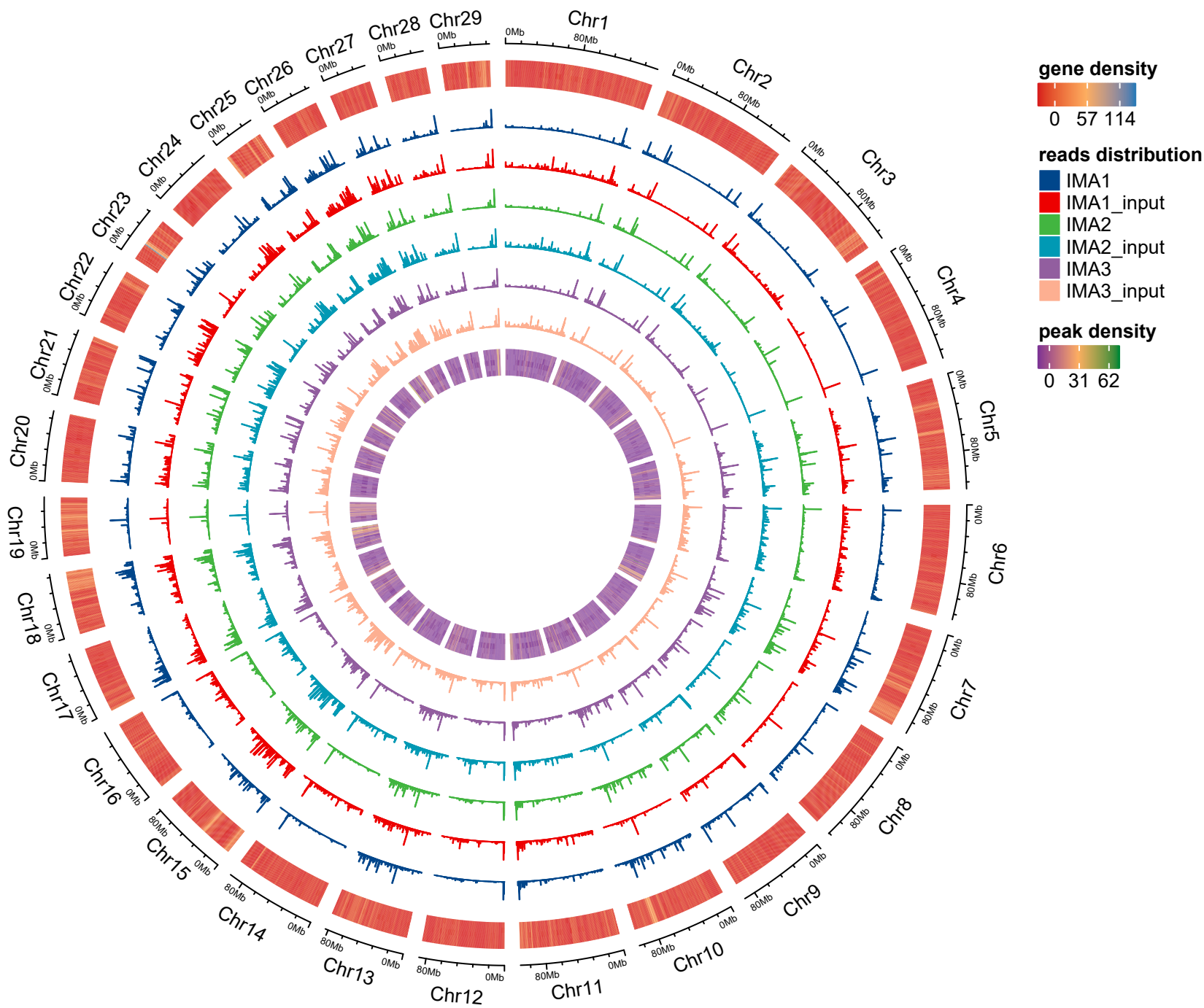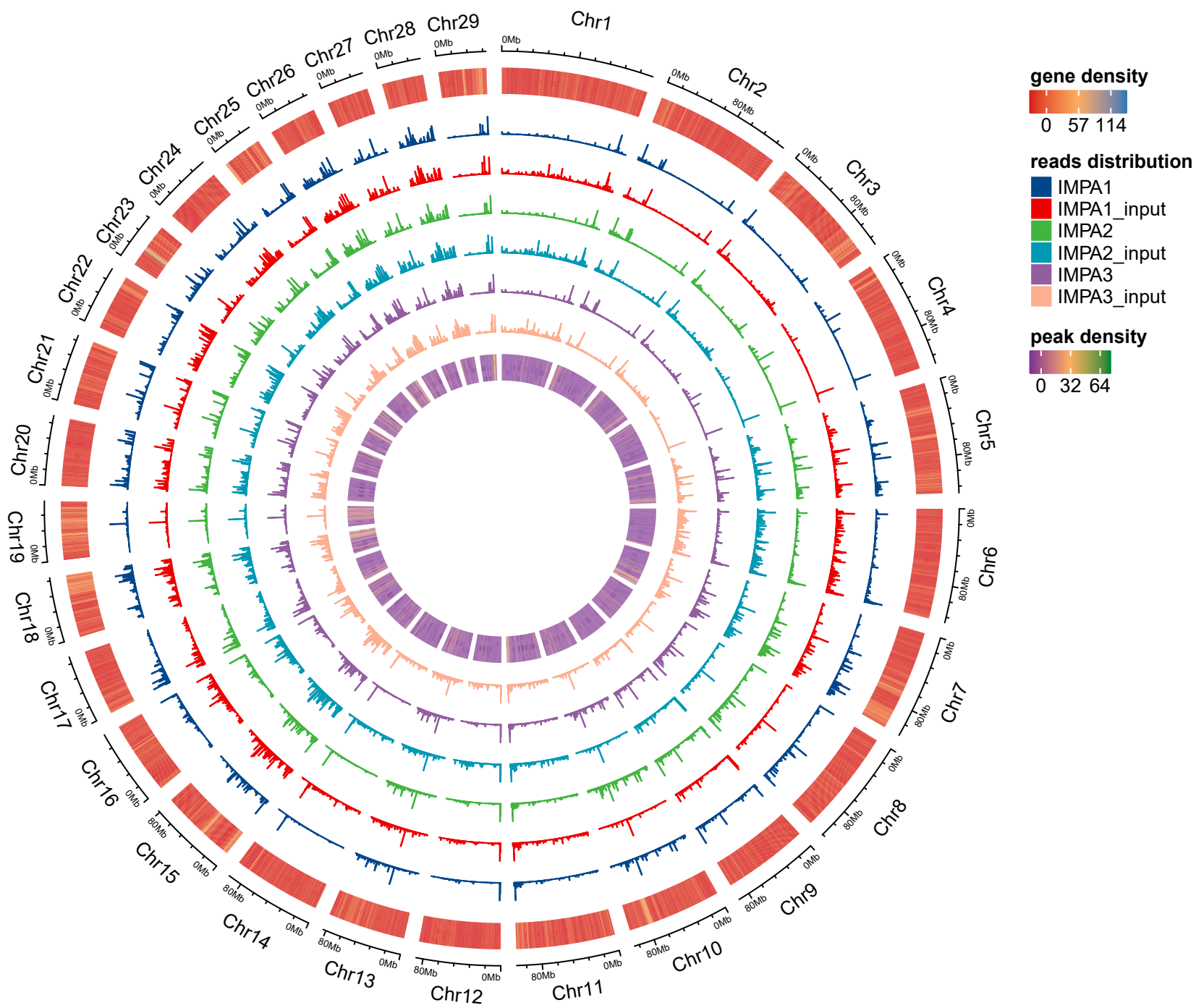

Supplement: Supplementary file 1 [file ijms-24-04817-s001.zip › Figure S1.pdf]

A

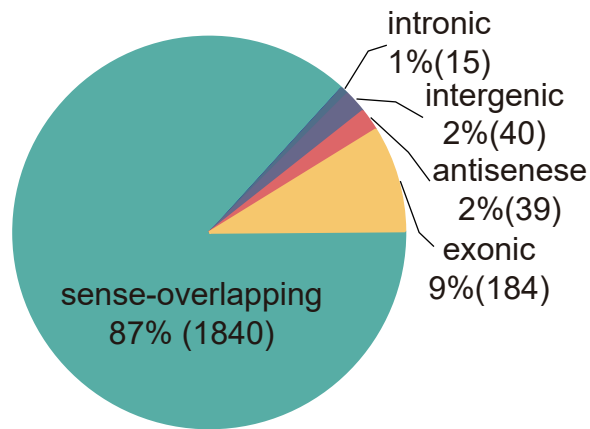

B

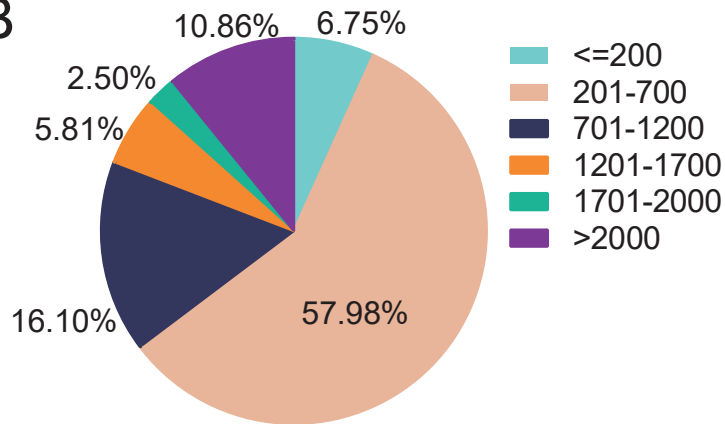

C

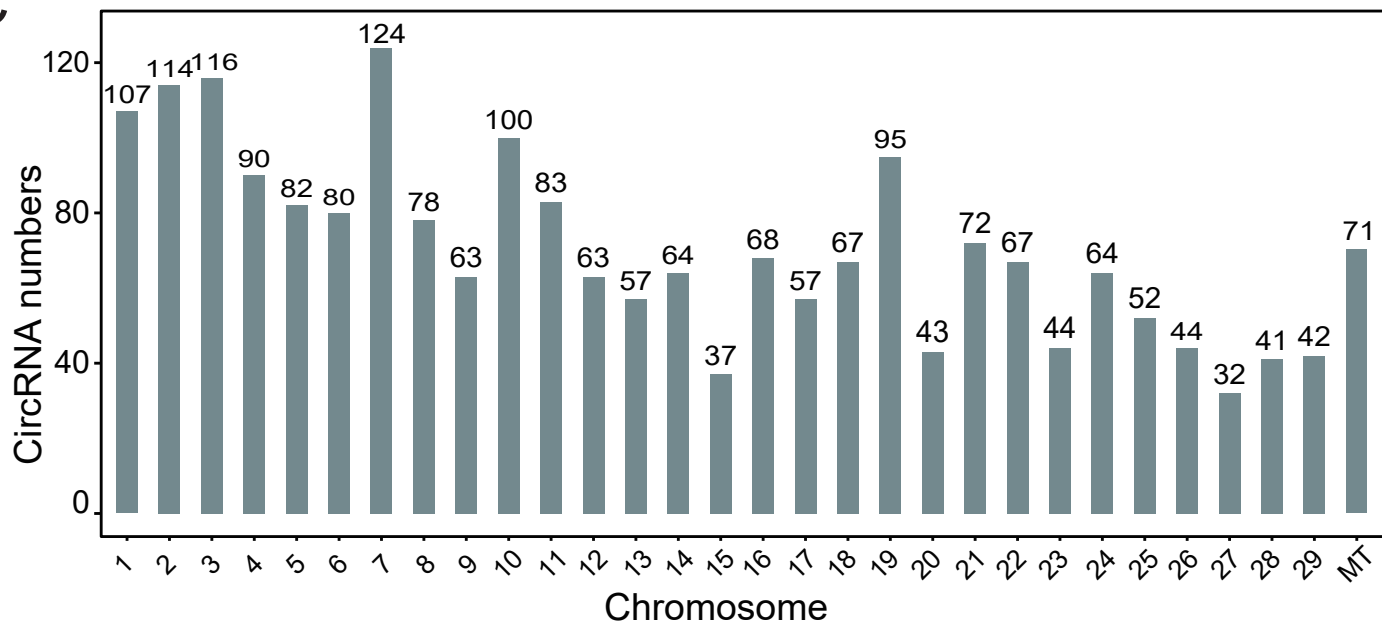

Supplement: Supplementary file 1 [file ijms-24-04817-s001.zip › Figure S2.pdf]

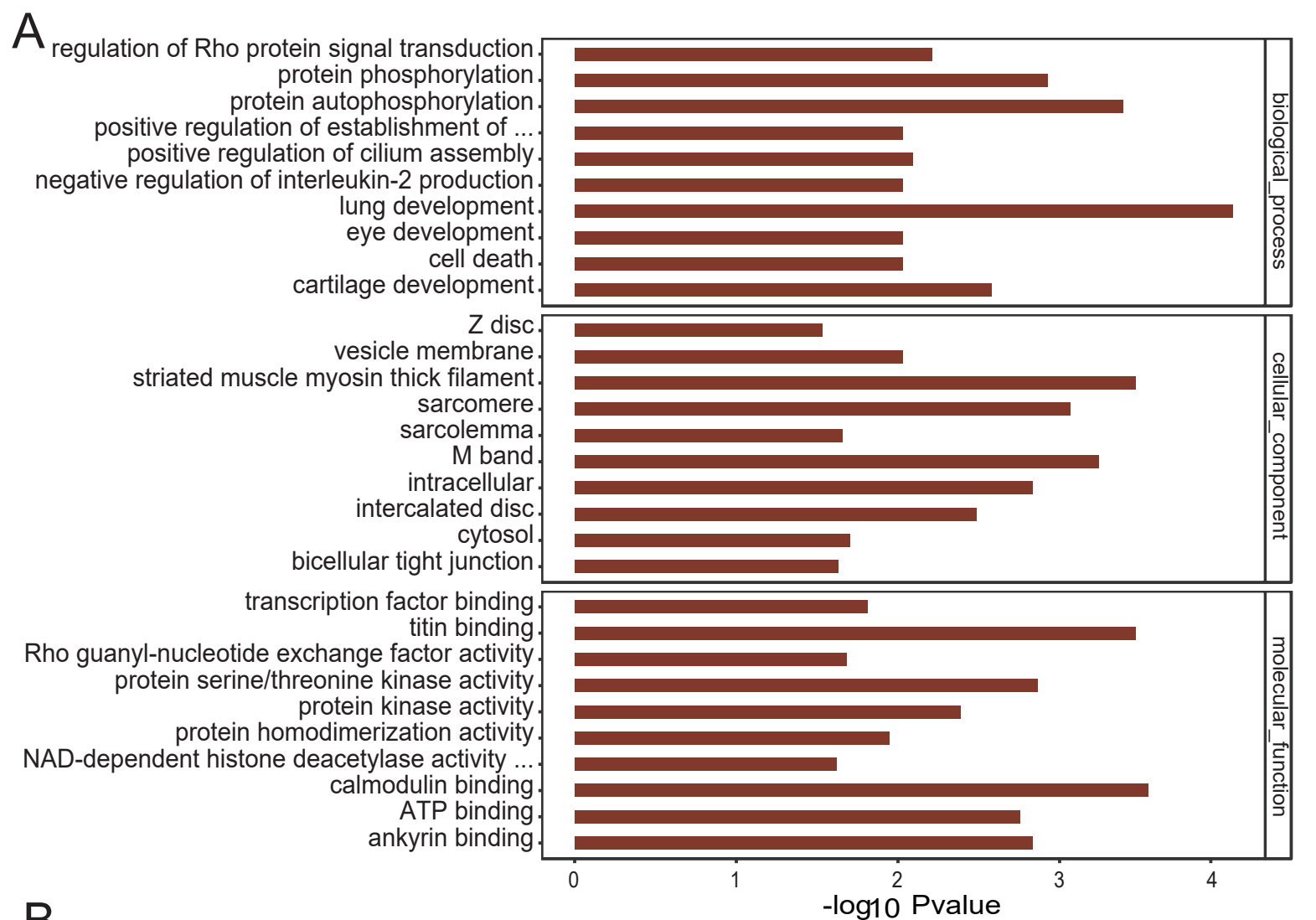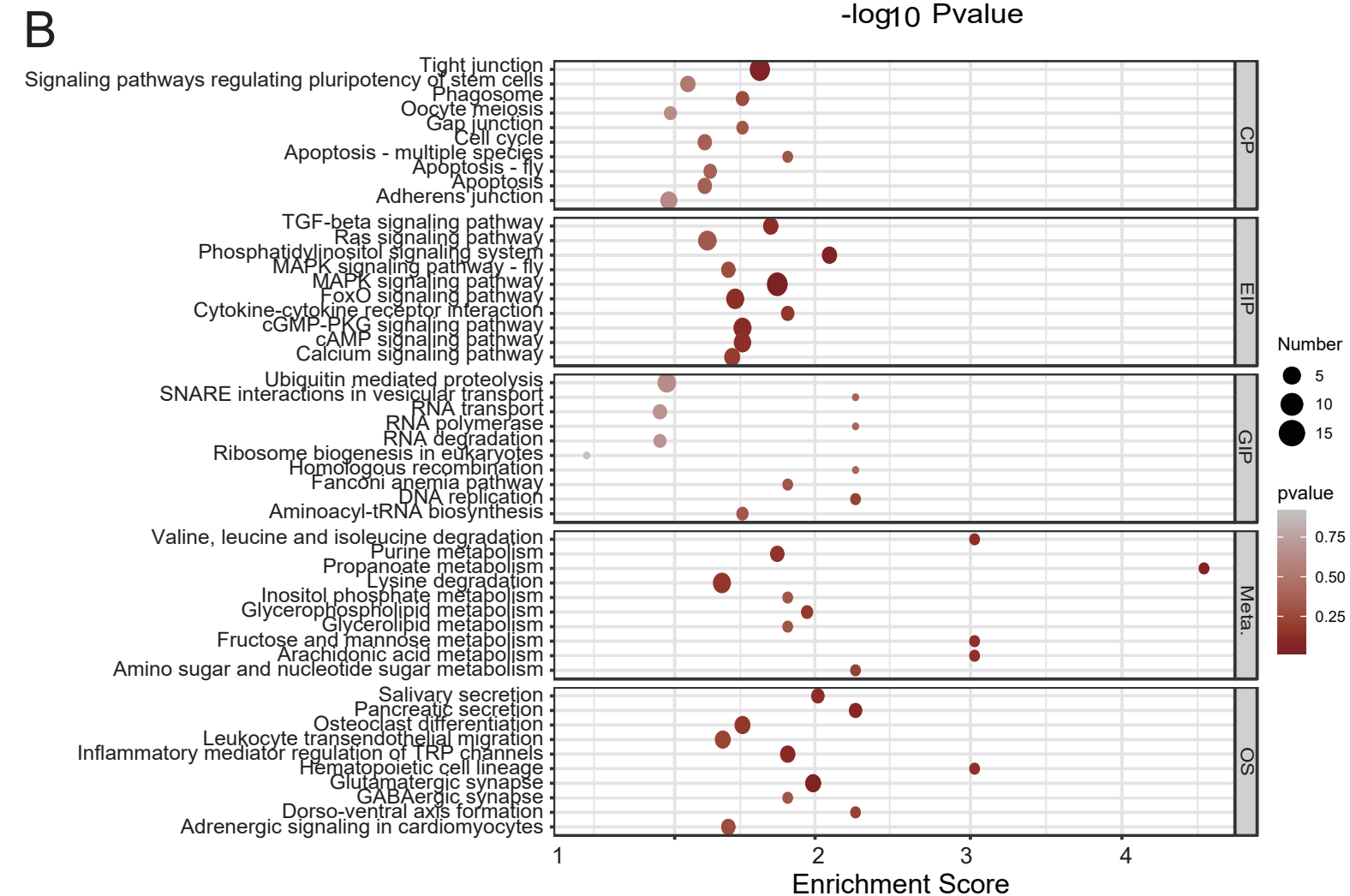

Supplement: Supplementary file 1 [file ijms-24-04817-s001.zip › Figure S3.pdf]
